# Examining the Effect of Polymer Extension on Protein–Polymer Interactions That Occur during Formulation of Protein-Loaded Poly(lactic acid-co-glycolic acid)-polyethylene Glycol Nanoparticles

**DOI:** 10.3390/polym14214730

**Published:** 2022-11-04

**Authors:** Chris W. Nyambura, Elizabeth Nance, Jim Pfaendtner

**Affiliations:** Department of Chemical Engineering, University of Washington, Seattle, WA 98195, USA

**Keywords:** molecular dynamics, nanomedicine, theory and modeling, drug delivery

## Abstract

Protein therapeutics have the potential to treat a wide range of ailments due to the high specificity in their function and their ability to replace missing or mutated genes that encode for key cellular processes. Despite these advantages, protein drugs alone can cause adverse effects, such as the development of cross-reactive neutralizing antibodies. Through the encapsulation of proteins into nanoparticles, adverse effects and protein degradation can be minimized, thus improving protein delivery to sites of interest in the body. Nanoparticles comprised of poly(lactic acid-co-glycolic acid)-polyethylene glycol (PLGA-PEG) diblock copolymer are promising protein delivery systems as they are well characterized, non-toxic, and biocompatible. Desirable nanoparticle characteristics, such as neutral surface charge and uniformity in size and dispersity, can be achieved but often require the iterative manipulation of formulation parameters. Chain conformations in the formulation process are very important, and determining whether or not an extended or semi-collapsed polymer chain in the presence of a protein results in more favorable binding has yet to be investigated experimentally. Therefore, this work used atomistic molecular dynamics to examine the role of polymer extension on protein binding and its impact on the encapsulation process within PLGA-PEG nanoparticles. Three polymers (PLGA-PEG, PLGA, and PEG) were evaluated and iduronate-2-sulphatase (ID2S) was used as a model protein. We found highly expanded PLGA-PEG conformations led to more favorable binding with ID2S. Furthermore, PEG oligomers were observed to undergo transient binding with ID2S that was generally less favorable when compared to the other polymer types. The results also suggest that the relaxation times of the PLGA homopolymer and the PLGA-PEG copolymer at different molecular weights in relevant solvent mediums should be considered.

## 1. Introduction

Protein therapeutics have the potential to address pressing challenges facing human healthcare today. When compared to small-molecule drugs, this class of macromolecules can provide high specificity, can act as mediators in most intercellular pathways, and can replace critical proteins in individuals missing necessary or mutated genes that encode key cellular processes [1,2]. Despite these advantages, the delivery of protein drugs in free form is limited; their intravenous or subcutaneous administration can include protease recognition and cleavage once in the bloodstream, the development of cross-reactive neutralizing antibodies, and an increased risk of injection-site or viral infections [3,4,5]. Nanocarriers can address issues in protein delivery by preserving structure in proteolytic conditions and reducing opportunities for drug opsonization, allowing for sustained release and improving delivery [6]. Polymeric nanoparticles (PNPs) comprised of poly(lactic acid-co-glycolic acid)-polyethylene glycol (PLGA-PEG) are non-toxic, biodegradable, and biocompatible, and are well suited for various biomedical applications [7,8]. Using PLGA-PEG allows for desirable nanoparticle characteristics, such as neutral surface charge and uniformity in size and dispersity, typically achieved using emulsion-based or solvent evaporation methods, but only after the iterative manipulation of formulation parameters. A lack of molecular-scale details driving the formation of monodisperse and highly loaded PLGA-PEG nanoparticles leads to trial-and-error work in the development of new formulations. Furthermore, copolymer chains in solution fluctuate between extended or collapsed conformations, which can result in a wide range of protein-polymer (prot-poly) binding modes that may dictate the overall nanoparticle’s protein loading and release profile. With advances in computational capacity over recent years, knowledge of the most favorable binding modes between the polymer and protein can be garnered from computational tools, such as atomistic molecular dynamics (MD) simulations, and allow for the bottom-up design of protein-loaded PLGA-PEG nanoparticles.

Prior experimental and computational studies examining protein–polymer interactions in various drug delivery and biopharmaceutical applications have provided useful examples for how researchers can connect atomic-level observations to those seen on the mesoscale. Scanning transmission X-ray microscopy, employed by Leung et al., to investigate human serum albumin (HSA) adsorption on phase-separated polystyrene-b-poly(methyl methacrylate) (PS-b-PMMA) thin films, showed increased adsorption of HSA near inter-domain interfaces between PS and PMMA, in addition to other tested proteins showing preferred adsorption on hydrophobic PS domains [9]. Computationally, Yang et al., simulated an insulin–water–PEG system while varying polymer chain length by using a simulated annealing procedure to achieve faster convergence on the system’s equilibrium state for high PEG molecular weights [10]. After an equilibrium state was reached, they ran the systems for 10 nanoseconds (ns) and found that the PEGylated insulin structure was similar to that of free insulin. Moreover, they observed the occurrence of favorable hydrophobic interactions with the insulin surface at certain PEG chain lengths, likely resulting in a decrease in activity at high PEG molecular weight. Previous work from Nyambura et al., characterized polymer structure properties of PLGA-PEG and its homopolymer constituents in solvents ubiquitous to the aforementioned formulation methodologies [11]; however, no study investigated whether or not varying a polymer’s radius of gyration results in more favorable protein drug binding and influences the driving forces necessary for high protein drug loading. Ultimately, combining atomistic MD and experimental studies, like those described by Leung et al., will be critical to develop a deeper mechanistic understanding of dominant protein–polymer interactions during the formulation of PNPs.

In this work, protein–polymer interactions were evaluated by simulating three polymer oligomers in the presence of a therapeutically relevant protein, iduronate-2-sulphatase (ID2S), and pure water. This MD model was based on the experimental polymer and protein concentrations used in formulation methodologies employing PLGA-PEG. ID2S is a protein of interest because it is the main cause of Hunter’s syndrome, an inherited genetic disorder that results in the accumulation of glycosaminoglycans within lysosomes, due to ID2S deficiency [12]. Therefore, ID2S-loaded PLGA-PEG nanoparticles can potentially be used as an enzyme replacement therapy, thus providing patients with a viable treatment option for this rare disease. Three polymers (PLGA-PEG, PLGA, and PEG) and three levels of oligomer extension (high, medium, and low) were tested to observe if there are differences in copolymer–protein interactions when compared to its homopolymer constituents, in addition to understanding whether polymer conformation has a large or negligible impact on protein–polymer interactions. We aim to use insights from this investigation to explain how polymer conformation, which is controlled by the choice of the organic solvent, affects protein–polymer interactions that occur at either the first step of the nanoparticle formulation process when polymer organic solution is mixed with protein aqueous solution, the very early stages of nanoparticle formation when the protein-polymer mixture is added dropwise to a relatively large water-surfactant sink, and during protein release out of PLGA-PEG nanoparticles while dissolved in an aqueous medium.

## 2. Materials and Methods

### 2.1. Simulation Details

GROMACS 2020.5 [13,14] was used to simulate all ID2S/polymer systems in the NPT ensemble at 298 K, 1 bar, and in individual water–ion medium simulations of 200 ns in length. The ID2S structure was taken from the RCSB protein database (PDB code 5FQL). Using the Pymol [15] and Modloop [16] online servers, a missing loop region was reconstructed from the FASTA sequence, and then energy minimization was performed to remove steric clashes among neighboring residues. Furthermore, ID2S was simulated without the C-terminus tail present in the FASTA sequence to ensure protein stability over the course of the MD simulation. The AMBER19SB forcefield [17] was used for ID2S partial charge and topological parameters, while the general amber forcefield [18,19] was used for the polymers’ topological parameters. AmberTools21 [20] and ParmEd [21] were used to generate and convert amber files to gromacs compatible topology files. Partial atomic charges for each polymer type were ascertained through the residual electrostatic potential fitting method, using the Hartee–Fock level with the 6–31 G(d) basis set in Gaussian 09 [22], similar to our previous work by Nyambura et al. [11]. Charges of individual monomer units and capping units were all scaled to have a net charge of zero to ensure scalability to different oligomer lengths without reparameterization. The OPC solvent model [23,24] was used for explicit water, while temperature control was achieved using the Bussi–Donadio–Parinello thermostat [25], and pressure control was achieved using the Parrinello–Rahman barostat [26]. In order to maximize computational efficiency, the hydrogen mass repartitioning method [27] was used to allow for a 4-femtosecond timestep.

**Figure A1:**
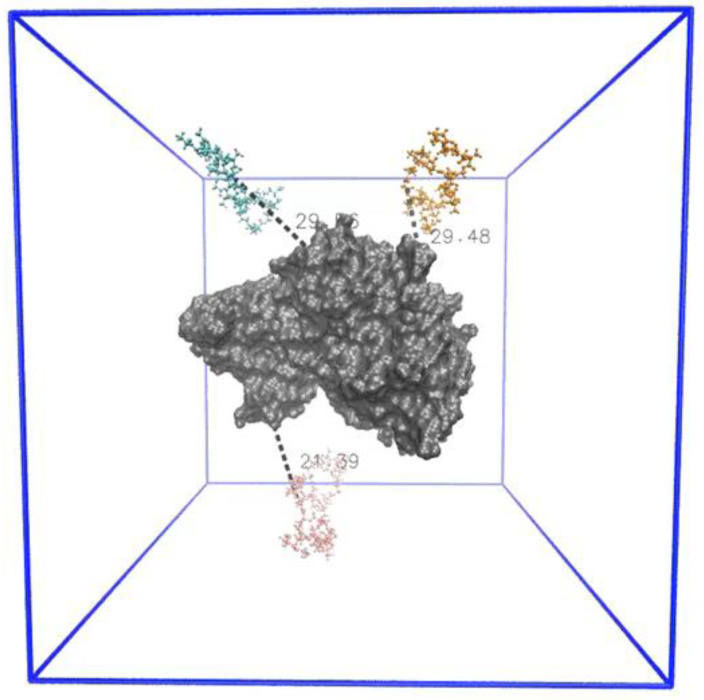
An example of an initial configuration of a polymer/ID2S simulation box, whereby oligomers are ~2–3 nm away from the protein surface. Different simulation trials were generated using PACKMOL by placing polymer oligomers in different areas within the simulation box, while maintaining a 3 nm distance.

All systems were packed within a cubic box with a side length of 11 nanometers (nm) using PACKMOL [28]. ID2S had a net charge of −20, while the tested polymers had a zero net charge; hence, 20 sodium ions were added to each simulation to ensure charge-neutral conditions. A harmonic potential, with a scaled force constant (*k*) of 140 kJ/mol, was used to restrain the polymer oligomers at a certain value of radius of gyration by utilizing the Colvars module within GROMACS 2020.5 [29]. Each simulation, following the packing of 3 polymer oligomers, 1 ID2S protein, water, and sodium ions into a cubic box, first underwent a steepest descent energy minimization step and two equilibration periods at constant volume and temperature (NVT), followed by constant pressure and temperature (NPT), all while restraining the polymer and protein chains. The NVT and NPT equilibration period was set at 2 ns for all simulations. An example of an initial configuration can be seen in Figure A1, whereby polymer oligomers were placed 2–3 nm away from the ID2S surface to allow for a proper equilibration in the system, prior to protein–polymer contact. Coordinates, velocities, and energies were saved every 10 picoseconds. Short-range coulombic and van der Waals interactions employed the Verlet cutoff scheme, using a cut-off radius of 1 nm. Particle-mesh Ewald (PME) summation was used for long-range interactions, where the PME order was 4 and the Fourier spacing was 0.16. LINCS was used as the main constraint algorithms for bonds. MDAnalysis [30] and Plumed 2.7 [31,32,33] were the main packages used for analyzing all simulations, alongside in-house python codes.

### 2.2. The Design of Experiments and Simulation Protocol

**Figure 1:**
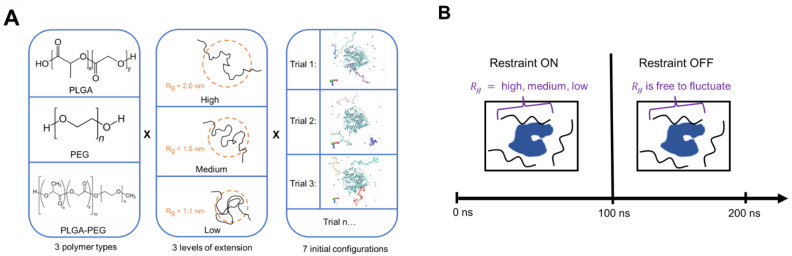
Layout of (**A**) the experimental design implemented in this study and (**B**) the progression of each simulation, where all oligomers in the simulation box are restrained to the same R_g_ value corresponding to a certain level of extension.

**Figure A2:**
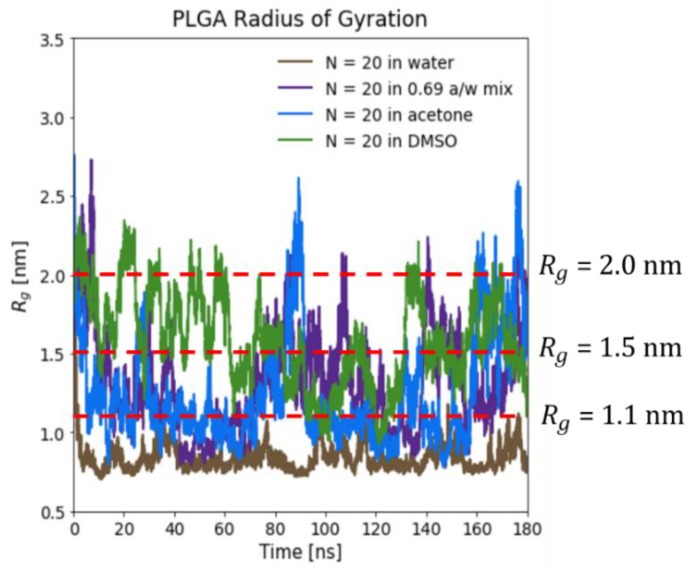
Radius of gyration as a function of simulation time is plotted above for PLGA oligomers at a monomer length of 20 (L_c_ = 10.4 nm) in various solvent conditions encountered during the formulation of PLGA-PEG nanoparticles. The red lines indicate the chosen R_g_ values used in this study, where oligomer R_g_ of 2 nm, 1.5 nm, and 1.1 nm correlate with high, medium, and low levels of extension, respectively.

To ensure the adequate sampling of different binding modes across 3 polymer types and 3 levels of extension, 7 different initial configurations were used, with the oligomers randomly oriented around the protein across each trial; this resulted in 63 simulations in total (Figure 1A). A harmonic potential was used to restrain all 3 oligomers’ radius of gyration (R_g_) at high (2 nm), medium (1.5 nm), and low (1.1 nm) levels of extension for the first 100 ns (restraint-ON phase) to assess the effect of varying solvent quality on protein–polymer binding. Table 1 shows that, regardless of the polymer type, all simulated oligomers are nearly the same contour length (L_c_). By holding the L_c_ constant, the same R_g_ values corresponding to the three levels of extension could be used across the three tested polymers, since the range of possible oligomer sizes in solution, across polymer types, will approximately be the same and will allow for a more systematic investigation. These R_g_ values were based on classical simulations of PLGA oligomers in various solvents (Figure A2) ubiquitously used in nanoparticle formulations.

After 100 ns, the harmonic restraint was turned off (restraint-OFF phase) and the system was allowed to propagate for another 100 ns to gain some insights on whether polymer binding was irreversible in the pure water medium, mimicking the step where the protein–polymer mixture was added dropwise, and PLGA-PEG nanoparticle formation began. A total simulation time of 200 ns was chosen because the diffusion of ID2S through a ~10 nm box would last ~200–300 ns, calculated using the Stokes–Einstein diffusion equation to estimate the protein’s motion in pure water. Furthermore, prior investigations into the solvent displacement method show that the diffusion of water-miscible solvent and other molecular species within the non-solvent medium is rapid, when compared to the rate of nanoparticle nucleation [34,35]. Therefore, we assume that the pure water environment in the restraint-OFF phase closely mimics the early stages of ID2S/PLGA-PEG cluster formation.

### 2.3. The Characterization and Analysis of Protein–Polymer Interactions

Prior to investigating the molecular driving forces behind protein–polymer binding, the protein structure over the course of a MD simulation was examined to ensure that solvent-accessible residues primarily interact with the polymer. The root-mean-squared deviation (RMSD) of a protein’s backbone from its crystal structure, ascertained from the RCSB protein databank, was the main metric used in simulations containing a protein. N is the total number of backbone heavy atoms, where δ_i_ indicates the distance between atom i and its reference position within the crystal structure (Equation (1) below). Furthermore, all simulations containing a protein were simulated within a pure water medium only since the AMBER19SB forcefield was optimized to reproduce experimental protein structures in aqueous environments [17].

(1) $$\mathrm{RMSD}=\sqrt{\frac{1}{N}\overset{N}{\underset{i=1}{\sum }}\delta_{i}^{2}}$$

**Figure 2:**
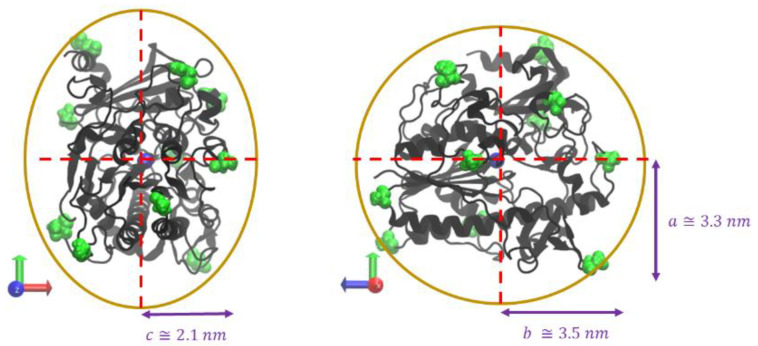
Iduronate-2-sulfatase (ID2S) approximated ellipsoidal shape where a ≅ 3.3 nm, b ≅ 3.5 nm, and c ≅ 2.1 nm. The blue sphere in the center of ID2S is the protein’s center of mass and surface residues colored in green are surface residues used to determine the semi-axial lengths.

Next, the distance between the center of mass (COM) of the protein and each polymer oligomer ($d_{i}^{\mathrm{COM}}$, where i is the oligomer identifier) was used to understand the general extent of protein–polymer interactions, over the course of a simulation trajectory. Similarly, the distance between the COM of each oligomer to another oligomer ($d_{i-j}^{\mathrm{COM}}$, where i is the oligomer identifier and j is the next oligomer where i ≠ j) was examined to understand the extent of polymer–polymer interactions. These distances, over simulation time, were extracted using the open-source community-developed PLUMED library [31,32] (version 2.7.1), and used to establish four classes of interactions: protein–polymer interactions only, polymer–polymer (poly–poly) interactions only, both protein–polymer and polymer–polymer (both prot–poly and poly–poly) interactions, and free in solution (free in soln.). Prot–poly interactions only were defined as $d_{i}^{\mathrm{COM}}$ ≤ 4.3 nm, due to the ID2S general shape and length of simulated oligomers. For example, ID2S shape, as seen in Figure 2, was approximated as an ellipsoid by measuring the distance between the protein’s COM and selected surface residues, whereby the largest semi-axial length was ~3.5 nm. Observations of multiple trajectories and an examination of different cutoff distances showed that 4.3 nm is a reasonable distance because it accounts for transient prot–poly interactions that can occur between different parts of individual oligomers and ID2S.

Poly–poly interactions only were defined as $d_{i-j}^{\mathrm{COM}}$ ≤ 2.3 nm to account for interactions that occur over a wide range of oligomer conformations; collapsed conformations, at the contour lengths present in this study, were measured to have R_g_ values less than 1.2 nm, whereas extended conformations were measured to have R_g_ values greater than 1.8 nm. In addition, initial configurations placed oligomers a distance greater than 3 nm away from each other, similar to Figure A1. Hence, this cutoff distance was chosen to ensure different interaction modes between polymer oligomers are being captured across the different R_g_ values tested. Both prot–poly and poly–poly interactions only were defined when $d_{i}^{\mathrm{COM}}$ ≤ 4.3 and $d_{i-j}^{\mathrm{COM}}$ ≤ 2.3 nm, whereas free in solution was defined when neither of those two distance cutoffs were met. By categorizing simulation trajectory observations into these 4 interaction classes, the general interplay between protein–polymer and polymer–polymer binding can be better understood, providing a path to determine the most favorable protein–polymer interactions for the purpose of protein loading within PLGA-PEG nanoparticles. To understand how often each protein–polymer simulation spent in each of the 4 interaction classes, the time frequency (TF) of each oligomer’s interaction class was calculated, and used to examine how trial simulations with different initial configurations explored phase space. As seen in Equations (2) and (3), i is the oligomer identifier and N_olig_ is the total number of oligomers. For each simulation trial, this metric was extracted for each oligomer and averaged (${\mathrm{TF}}_{\mathrm{avg}}$) to allow for easy data visualization and aid analysis. ${\mathrm{TF}}_{\mathrm{avg}}$ values across simulation trials with the same polymer type were averaged again to evaluate the differences in protein binding across polymers with different chemical moieties (Equation (4)).

(2) $${\mathrm{TF}}_{i}=\frac{\begin{array}{c} \mathrm{No}.\;\mathrm{of}\;\mathrm{frames}\;\mathrm{oligomer}\;i\;\mathrm{spent}\\ \mathrm{in}\;a\;\mathrm{specific}\;\mathrm{interaction}\;\mathrm{class} \end{array}}{\mathrm{No}.\;\mathrm{of}\;\mathrm{frames}\;\mathrm{in}\;a\;\mathrm{simulation}\;\mathrm{trajectory}}$$

(3) $${\mathrm{TF}}_{\mathrm{avg}}=\frac{\sum_{i=1}^{N}{\mathrm{TF}}_{i}}{N_{\mathrm{olig}}}$$

(4) $${\mathrm{TF}}_{\mathrm{avg}}^{\mathrm{poly}}=\frac{\sum_{i=1}^{N_{\mathrm{trials}}}{\mathrm{TF}}_{\mathrm{avg}}^{i}}{N_{\mathrm{trials}}}$$

The strength of binding between ID2S and different polymer oligomers was examined using the GROMACS 2018.5 analysis toolkit [36] to calculate the short-range energetic contributions of various nonbonded interactions. Typically, London dispersion and repulsive forces are captured by the Lennard–Jones potential energy function, while electrostatic forces are described by Coulomb’s law. This information is valuable because it can show whether protein–polymer binding is more favorable than polymer–polymer binding, when polymer types varied or oligomers conformed. In addition, results from this analysis can help to explain why a certain protein–polymer interaction interface undergoes more irreversible binding events across simulation trials.

In order to understand the residence time of polymer oligomers near a set of protein surface residues over the course of a simulation, the percent occupancy (% occupancy) was calculated for each amino acid residue by counting the number of frames a polymer oligomer was within 4 angstroms (Å) of a protein amino acid surface amino acid, divided by the total number of frames within the entire trajectory (Equation (5)).

(5) $$\%\;\mathrm{Occupancy}=\frac{\begin{array}{c} \mathrm{No}.\;\mathrm{of}\;\mathrm{frames}\;a\;\mathrm{polymer}\;\mathrm{oligomer}\;\mathrm{was}\\ \mathrm{within}\;4\;Å\;\mathrm{of}\;\mathrm{an}\;\mathrm{AA}\;\mathrm{residue} \end{array}}{\mathrm{No}.\;\mathrm{of}\;\mathrm{frames}\;\mathrm{in}\;a\;\mathrm{simulation}\;\mathrm{trajectory}}\times$$

The percent occupancy for each amino acid was used to define a protein–polymer interaction interface from each simulation. Since some trials can result in unique binding modes, the prot–poly interface from each trial can be aggregated into a collapsed interface, for each polymer type and level of extension, by sorting repeating and non-repeating residues with non-zero occupancies into a new set of residues, in addition to averaging occupancy values for repeating residues. This was carried out using Python 3.6 [37], similar to most analyses shared in this work, and allowed for a more streamlined analysis workflow since binding zones from multiple trials can be collapsed onto a single interface. Subsequently, the elimination of low-occupancy residues can be performed to identify sets of surface amino acids with long polymer residence times. The cutoff values of percent occupancy that were used to eliminate amino acids (AA) with low occupancy values and determine amino acids with high residency ranged between 50% and 90%. Residues with occupancy values greater than 50% were observed to undergo favorable binding with the simulated polymer, while also accounting for reversible binding events. Residues with occupancy values greater than 90% allow specific residues with experienced irreversible binding to be identified, but ignore the local chemical surface environment near high-residency amino acids that likely contribute to protein–polymer binding. Once these filtered residues were extracted, depending on the cutoff occupancy value, they could be grouped into 5 categories: negative, positive, polar, hydrophobic, and aromatic. The number of residues in each grouping was then divided by the total number of residues with occupancy values greater than the cutoff, in order to understand the AA distribution of the sub-selected protein–polymer interface (Equation (6)).

(6) $$\mathrm{Fraction}\;\mathrm{of}\;\mathrm{residues}\;w/\;>\;X\%\;\mathrm{occupancy}=\;\;\frac{\begin{array}{c} \mathrm{No}.\;\mathrm{of}\;\mathrm{residues}\;\mathrm{in}\;\mathrm{an}\;\mathrm{AA}\;\mathrm{grouping}\\ \mathrm{with}\;>\;X\%\;\mathrm{occupancy} \end{array}}{\mathrm{Total}\;\mathrm{no}.\;\mathrm{of}\;\mathrm{residues}\;\mathrm{with}\;>\;X\%\;\mathrm{occupancy}}$$

Filtering surface amino acids, via the calculation of percent occupancies, is useful for identifying a binding interface but does not consider the effect of residue clusters on protein–polymer interactions. Knowledge of the local chemical environment near high-residency amino acids is desired because the rational design of protein-loaded nanoparticles requires such information to have precise control over protein loading and release. However, methodologies to ascertain such information have varied greatly, and no standard has been established to systematically perform such an investigation [38]. Taking inspiration from Jones et al. [39] in their analysis of interaction sites within protein–protein complexes, a surface patch analysis methodology was used to more strictly examine the collapsed protein–polymer interface and determine: (1) what makes this interface different from the rest of the protein surface and (2) the AA composition and average number of residues per cluster involved in irreversible polymer binding. Surface patch analysis applied to protein–polymer simulations consisted of generating n overlapping, contiguous surface patches with a varying number of surface amino acids. Surface residues were determined using the VMD plugin [40] to measure the solvent-accessible surface area (SASA) of all residues within a given protein. Residues with a SASA value greater than 1 Å^2^ were considered surface residues because water molecules have an approximate radius of 1.4 Å [41], and are likely to interact with these selected amino acids. Moreover, these residues were used to calculate the protein–polymer contact surface area (CSA), as seen in Equation (7) below, where N_interface_ is the number of residues in the protein–polymer interface and SASA_AA,i_ is the SASA value of an amino acid in the interface.

(7) $$\mathrm{Protein}–\mathrm{Polymer}\;\mathrm{CSA}=\sum_{i=1}^{N_{\mathrm{interface}}}{\mathrm{SASA}}_{\mathrm{AA},i}$$

**Figure A3:**
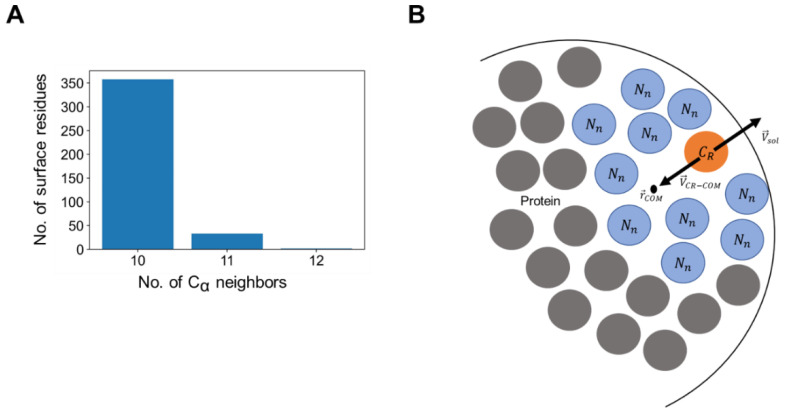
(**A**) Using the MDAnalysis Neighbor Search Wrapper with a search radius of 10 Å, the number of surface residues with 10, 11, or 12 C_α_ atom neighbors, for ID2S, was calculated and plotted. (**B**) Schematic showing the determination of a solvent vector (${\vec{V}}_{\mathrm{sol}}$) for each surface residue. Each sphere represents a C_α_ atom of a surface residues. C_R_ is the central surface residue whose solvent vector is being calculated and *N_n_* is the *n* nearest atom neighbor. ${\vec{r}}_{\mathrm{COM}}$ is the location of the center of mass of the n nearest neighbors and ${\vec{V}}_{\mathrm{CR}-\mathrm{COM}}$ is the vector used to define.

For each surface residue, a solvent vector (${\vec{V}}_{\mathrm{sol}}$) was calculated to ensure that residues on the opposite face or rings of residues were not selected in a surface patch. This vector is defined by first finding the C-alpha (C_α_) atoms of the *n* nearest surface residues, using a given surface amino acid C_α_ atom as the center of the search. MDAnalysis Neighbor Search Wrapper [30] was utilized to perform atom and residue nearest neighbor search. Specifically, the MDAnalysis search wrapper could not always provide the same number of nearest neighbors for each residue, since the user has to provide a range of radial values used by the algorithm to execute its search. However, a range of radial values were determined such that most surface residues had 10 nearest neighbors, with the remaining AAs having 11 or 12 near neighbors (Figure A3). Using these atoms, the COM was calculated and used to define a vector between the central surface residue and the COM of its n nearest neighbors (${\vec{V}}_{\mathrm{CR}-\mathrm{COM}}$); this vector was normalized to determine the surface patches. The inverse of normalized ${\vec{V}}_{\mathrm{CR}-\mathrm{COM}}$ results in ${\vec{V}}_{\mathrm{sol}}$ (Figure A3).

**Figure A4:**
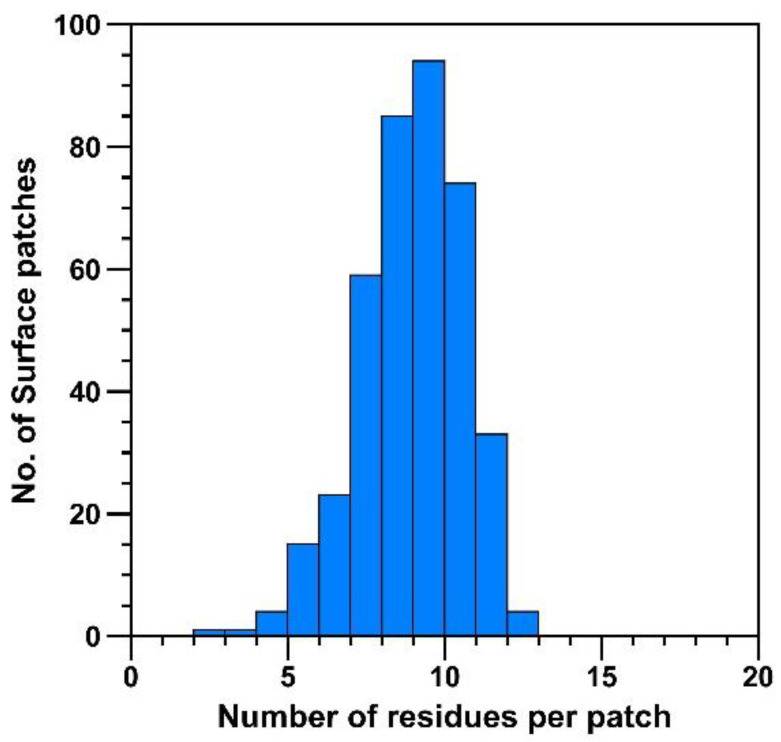
Number of residues per surface patch when the angle cutoff for adjacent residue inclusion within a surface patch was 125°.

**Figure A5:**
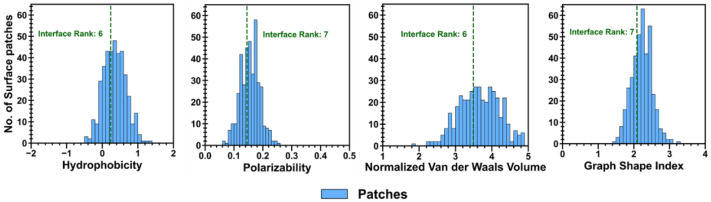
The distribution of the four physiochemical descriptors for ID2S surface patches (in blue) are plotted, in addition to the parameter ranking and descriptor values for a selected protein–polymer interface (green dashed line). For example, the hydrophobicity of the selected interface is ~0.22, meaning it is in the 60–70% range of the hydrophobicity patch distribution and therefore obtains a rank of 6. This comparison of the physiochemical descriptors between a sample interface and the generated surface patches was valuable in characterizing differences in protein–polymer binding.

To define an overlapping surface patch for each surface residue, a nearest residue neighbor search was performed on the surface amino acids, employing a search radius of 13 Å. This also resulted in the same distribution of nearest neighbors (Figure A3), where most surface residues also had 10 nearest residue neighbors. Using the solvent vectors and a given surface residue as the center of the patch, an adjacent nearest residue neighbor was included in a patch if the angle between its ${\vec{V}}_{\mathrm{sol}}$ and the central residue’s ${\vec{V}}_{\mathrm{sol}}$ was less than a chosen cutoff value (θ_cut_). For ID2S, θ_cut_ was chosen to be 125° after examining the resulting distribution of the number of residues per patch while varying θ_cut_. Furthermore, most surface patches with θ_cut_ = 125° consisted of 9 residues, varying in morphology and AA composition (Figure A4). Next, 4 physiochemical descriptors for all 20 natural amino acids, experimentally measured by Fauchere et al. [42], were used to characterize the physiochemical nature of each overlapping surface patch, namely hydrophobicity (HB), polarizability (PB), graph shape index (GSI), and normalized van der Waals volume (nVdW). HB is described using water–octanol partition coefficients and GSI encodes for complexity, branching, and symmetry, providing a measure of the steric influence for a given patch. PB is a measure of how easy a residue’s electron cloud can be distorted, due to the presence of induced or permanent charge dipoles. nVdW was useful for analyzing why an interface may experience more dispersion forces when comparing across polymer types, since residues with larger nVdW can lead to greater dispersion forces. Calculations of each surface patch physiochemical property are found in Equations (8)–(11), where N_r,patches_ is the number of residues in a surface patch. By using these patch properties that capture the local chemical nature of defined residue clusters and examining their overall distribution (Figure A5), the characterization of the collapsed protein–polymer interface can be performed and compared to the other surface patches.

(8) $${\mathrm{HB}}_{\mathrm{patch}\;i\;}=\frac{\sum_{i=1}^{N_{r,\mathrm{patches}}}\;{\mathrm{HB}}_{\mathrm{AA},i}}{N_{r,\mathrm{patches}}}$$

(9) $${\mathrm{PB}}_{\mathrm{patch}\;i}=\frac{\sum_{i=1}^{N_{r,\mathrm{patches}}}\;{\mathrm{PB}}_{\mathrm{AA},i}}{N_{r,\mathrm{patches}}}$$

(10) $${\mathrm{GSI}}_{\mathrm{patch}\;i}=\frac{\sum_{i=1}^{N_{r,\mathrm{patches}}}\;{\mathrm{GSI}}_{\mathrm{AA},i}}{N_{r,\mathrm{patches}}}$$

(11) $${\mathrm{nVdW}}_{\mathrm{patch}\;i}=\frac{\sum_{i=1}^{N_{r,\mathrm{patches}}}\;{\mathrm{nVdW}}_{\mathrm{AA},i}}{N_{r,\mathrm{patches}}}$$

The same physiochemical descriptors, calculated for each surface patch, were also extracted for each protein–polymer interface ascertained from a simulation trial; they were determined the same way as seen in Equations (8)–(11), where N_r,patches_ is replaced with N_interface_, i.e., the number of residues in the collapsed interface. These descriptors were ranked on a scale from 1 to 10, relative to the overlapping surface patches. A rank of 1 means the prot–poly interface scores in the highest 10% range of a physiochemical descriptor distribution of all surface patches, whereas a rank of 10 reveals the prot–poly interface scores in the lowest 10% range [39]. This ranking provides a way to compare a prot–poly interface to other surface patches and to other interaction interfaces (Figure A5). The distribution of parameter interface ranks from multiple simulation trials allows the physiochemical nature of the protein–polymer interface to be evaluated for a given polymer type and level of extension, as each protein–polymer simulation explores a new region of phase space. Furthermore, such information can be utilized to understand how polymer chemistry may result in an observed interface with a certain parameter rank. As for the unique collapsed interface, whereby residues with occupancy values greater than 50% were sub-selected, percent patch overlap was calculated to determine which surface patches had the largest intersection with the collapsed interface, which contains surface residues with high (>90%) occupancy. Moreover, the effect of residue clusters on polymer binding can be examined using this metric. Equation (12) shows the calculation, where N_r,patch i_ is the number of residues in surface patch *i*.

(12) $$\%\;\mathrm{Patch}\;\mathrm{Overlap}=\frac{N_{r,\;\mathrm{patch}\;i}\;\cap {\;N}_{\mathrm{interface}}}{N_{\mathrm{interface}}}\;\times \;100$$

## 3. Results and Discussion

### 3.1. Copolymerization and Extension Effects on Protein–Polymer Interactions

**Figure 3:**
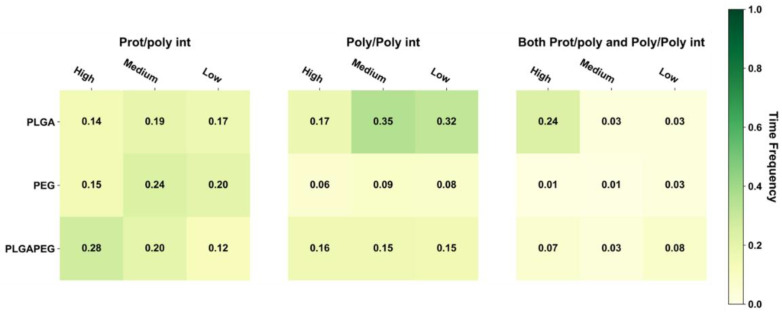
${\mathrm{TF}}_{\mathrm{avg}}^{\mathrm{poly}}$ values for each polymer type and interaction class, across different levels of oligomer extension. Time frequency values from simulation trials (n = 7) and oligomers (n = 3/trial) were averaged to arrive at the observed values above.

**Figure A6:**
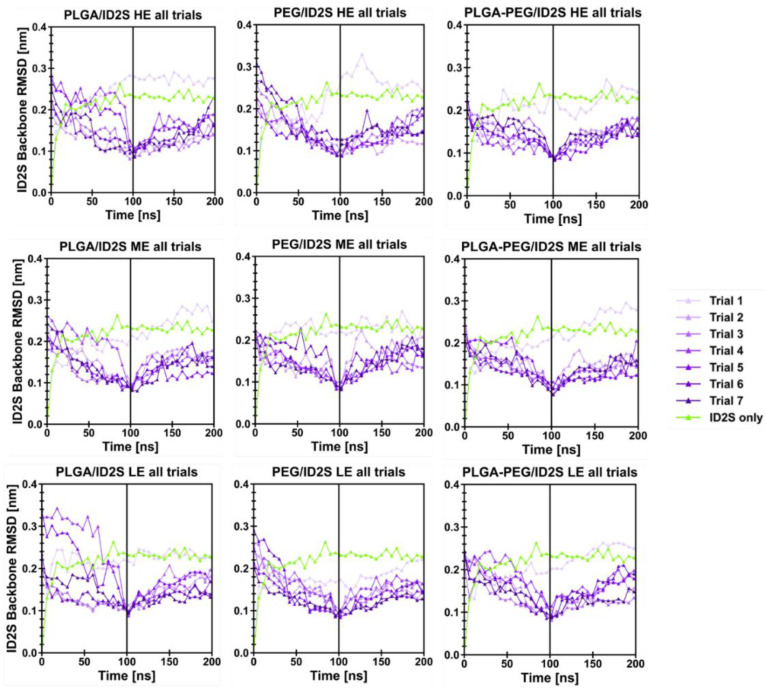
Root-mean-squared deviation (RMSD) of ID2S from its crystal structure versus time is shown for all levels of extension for PLGA/ID2S (**left** column), PEG/ID2S (**middle** column), and PLGA-PEG/ID2S (**right** column). RMSD values were plotted every 6 ns. ID2S in water system served as a control and is shown in green.

Protein stability across both restraint phases was evaluated by extracting ID2S backbone RMSD from each simulation trial as a function of time and comparing that time series to ID2S in water only (Figure A6). Across the three polymer types tested, RMSD fluctuations during all simulation trials were lower or similar to ID2S in water alone, showing that interactions between the protein and oligomers did not result in protein unfolding and that the simulated polymers primarily sample the ID2S surface. We visualized three of the four classes of interactions in Figure 3, whereby the oligomer-averaged and time-averaged time frequency (${\mathrm{TF}}_{\mathrm{avg}}^{\mathrm{poly}}$) for those three interaction classes can be seen for PLGA-PEG and its homopolymer constituents.

**Figure A7:**
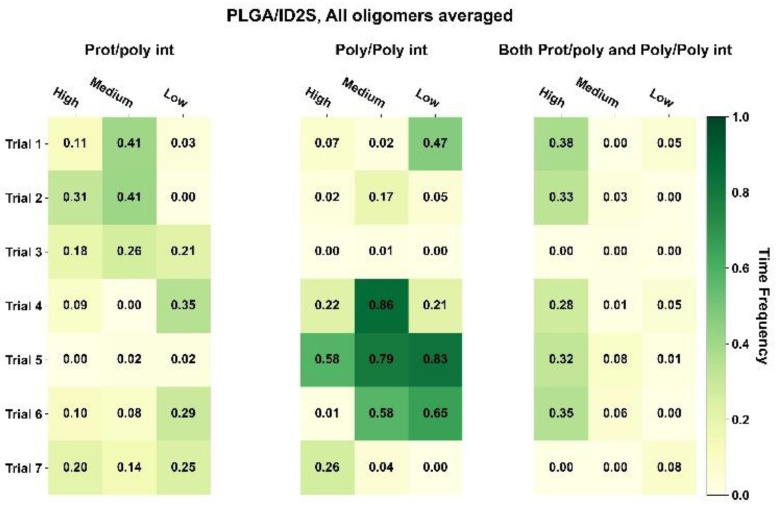

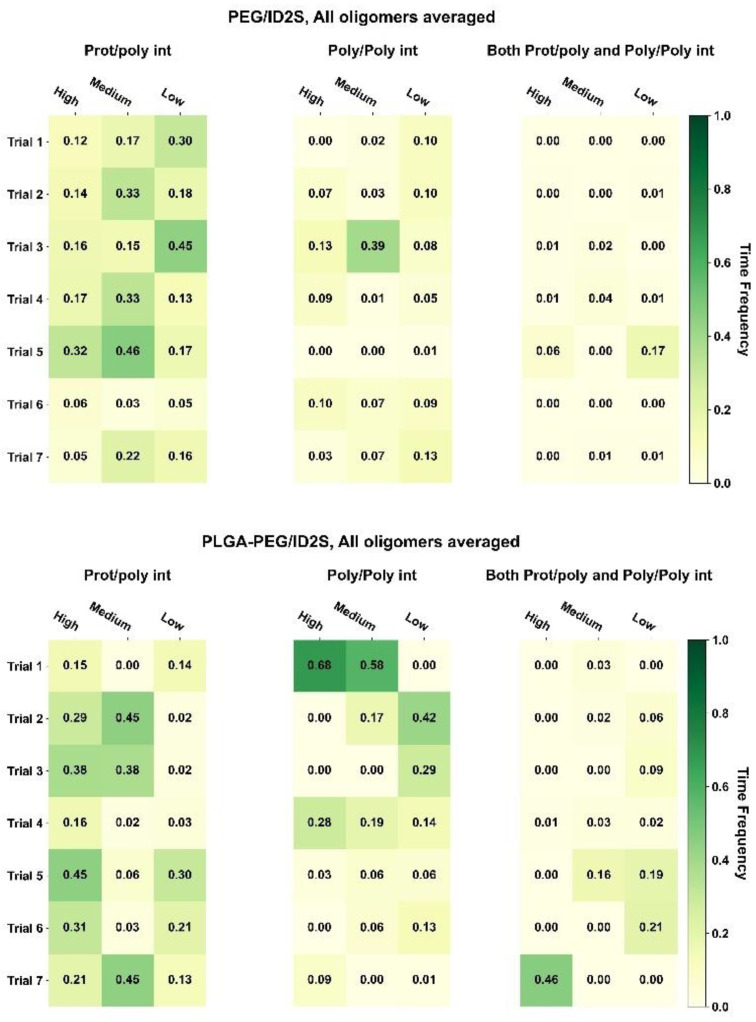
Oligomer-averaged time frequency of each interaction class, for each simulation trial across the different polymer types. Both restraint-on and -off phases were accounted for in the calculation of TF. These mean values were used to calculate ${\mathrm{TF}}_{\mathrm{avg}}^{\mathrm{poly}}$, as seen in the Results and Discussion section.

For PLGA/ID2S systems, poly–poly interactions occurred more often than prot–poly interactions for oligomers at medium and low levels of extension, whereas both prot–poly and poly–poly interactions were most frequent for the high extension systems. Moreover, prot–poly interactions only were still present for all levels of extension, showing that, in all system configurations, PLGA oligomers sampled the ID2S surface. An examination of TF across various configurations (Figure A7) at high and medium levels of extension shows that three of the seven trials (trials 4–6) underwent significant poly–poly interactions, indicating that polymer oligomers primarily aggregated and were free in solution at medium extension levels. This is due to PLGA oligomers existing in poor solvent conditions, resulting in more dominant LGA-LGA interactions. At low extension levels, trials 4 and 6 showed the highest frequency of prot–poly interactions only when compared to other trials, with little to no frequency observed for both prot–poly and poly–poly interactions across the seven trials. This suggests that, most times, collapsed PLGA oligomers would either individually contact the protein or be aggregated together in solution at low extension levels. At medium extension levels, trials 1–3 had a higher frequency of prot–poly interactions when compared to the other two classes, despite the driving force of PLGA oligomer aggregation; the effect of multiple trials is seen for PLGA/ID2S systems since four of the seven simulations were still able to undergo prot–poly binding without the oligomers rapidly aggregating.

As for the PEG/ID2S trials, poly–poly and both prot–poly and poly–poly interactions were minimal, where trials 3 and 5 were responsible for the TF values in these interaction classes. A medium level of extension was found to have the largest averaged TF value for the prot–poly interaction class, resulting from four trials experiencing favorable binding. This observation may be explained by the PEG oligomers being in good solvent conditions. At high extension levels, polymer–solvent interactions can occur more easily, and are likely to be more favorable than prot–poly interactions. The ease in which polymer–solvent interactions can occur, however, is reduced at medium and low levels of extension since opportunities for hydrogen bonding is limited; collapsed PEG oligomers still interact with ID2S, but TF values across trials were below 0.3 (6 of 7 trials), indicating that, for PEG/ID2S systems, the solvent had an appreciable impact on prot–poly interactions. Lastly, PLGA-PEG/ID2S systems showed that copolymerization reduces the extent of poly–poly interactions; furthermore, trial systems with oligomers at high extension levels resulted in more frequent prot–poly interactions (Figure A7), when compared to medium and low extension systems. Trials 5–7 had higher TF values for both prot–poly and poly–poly interactions, while trials 1–4 encountered more poly–poly interactions. Overall, increasing PLGA extension promoted more prot–poly interactions. When PLGA was copolymerized with PEG, poly–poly interactions were reduced, allowing favorable binding modes to be stabilized. During nanoparticle formulation, PLGA-PEG chains will have differing polymer blocks in their interaction with the solvent. This means that oligomers with collapsed LGA and extended EG domains may undergo less frequent protein contact, whereas extended LGA and EG domains result in more frequent protein contact.

**Figure 4:**
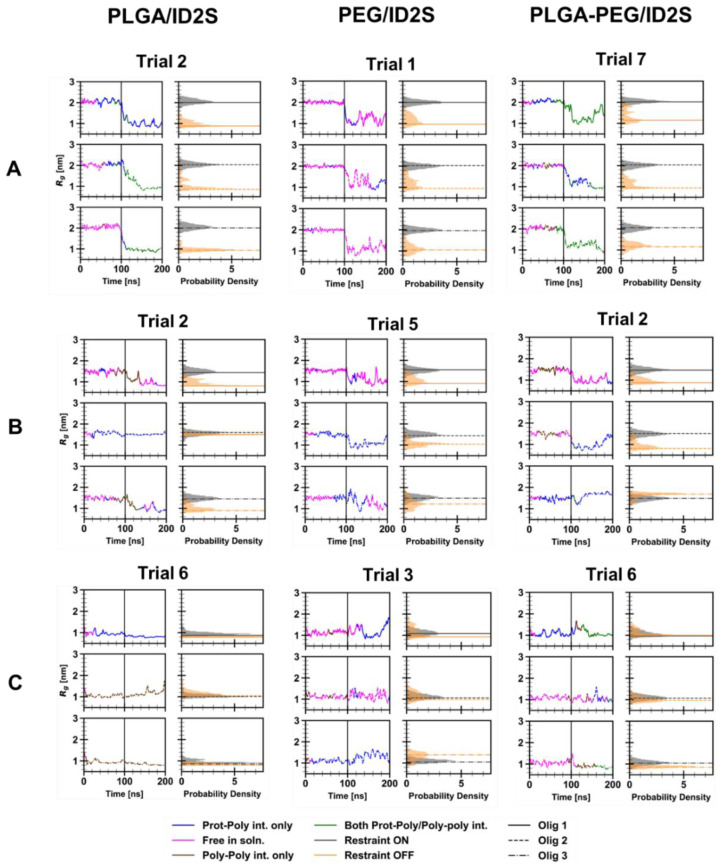
Radius of gyration (R_g_), following a moving average calculation to reduce noise, was plotted against simulation time for each oligomer for the selected trials, in addition to the probability density of R_g_ in both restraint-ON and -OFF phases. (**A**) indicates high extension, (**B**) medium extension, and (**C**) low extension for PLGA/ID2S (**left**), PEG/ID2S (**middle**), and PLGA-PEG/ID2S (**right**) systems. The four interaction classes were overlaid on the R_g_ vs. time data to understand the overall behavior of protein–polymer binding.

In order to understand how different levels of extension affects oligomer binding and extent of its interaction with ID2S, R_g_ time series and probability density data were extracted for each oligomer, as shown in Figure 4 for select simulation trials. Furthermore, the four interaction classes were overlaid over the R_g_ time series data to examine the transience of protein–polymer binding when the restraint was on or off. For the high extension case, oligomers in PLGA/ID2S trial 2 were initially free in solution, and then two oligomers encountered the ID2S surface separately when the restraint was on. Once the restraint was off, poly–poly interactions started to occur between the oligomers, while they were in contact with ID2S. One oligomer underwent rapid collapse, due to being in poor contact with ID2S, while the other two took 40–60 ns to collapse to 1 nm in size. This slow collapse can also be seen in a broader distribution of the probability density for those oligomers. This behavior was also observed in other trials whereby favorable PLGA oligomer binding with ID2S in the restrained phase resulted in the slow decay of R_g_ when the restraint was turned off. In addition, poly–poly interactions in early simulation times impacted the frequency of prot–poly interactions since oligomer aggregation persisted in some trials during the restrained phase and subsequently collapsed together while contacting the protein or being free in solution.

PEG/ID2S trial 1 oligomers at high extension levels showed reversible protein binding in both restrained and unrestrained phases. Probability densities of the oligomers across all trials were broader when compared to PLGA/ID2S trials after the restraint was turned off, indicating their return back to ideal-chain conformations due to being in good solvent condition. Across PEG/ID2S trials, regardless of the extension level, polymer–polymer interactions were infrequent, and oligomers were mostly free in solution in both phases; TF values extracted across trials also confirmed this observation of PEG/ID2S binding (Figure A7). Trial 5 at medium extension levels and trial 3 at low extension levels, however, exhibited irreversible binding between ID2S and one oligomer, showing that favorable PEG-ID2S interactions occur despite the strength of PEG–water interactions. Two oligomers in PLGA-PEG/ID2S trial 7 at high extension levels were mostly free and experienced transient prot–poly and poly–poly interactions in the restraint-ON phase, whereas one oligomer underwent irreversible binding. In the unrestrained phase, poly–poly interactions increased, while all three copolymer oligomers contacted the ID2S surface, resulting in a broader range of Rg values similar to the PEG/ID2S systems. Four trials showed irreversible binding between ID2S and at least one oligomer in the restrained phase and persisted in the unrestrained phase, with poly–poly interactions occurring less frequently than PLGA/ID2S systems. Overall, reducing polymer–polymer interactions between PLGA domains during the early stages of nanoparticle formation will likely be critical to promoting protein–polymer contact; however, strong polymer–solvent interactions would also not desired, as seen with the PEG/ID2S systems, because this leads to more reversible polymer binding events. Through PLGA copolymerization to PEG, poly–poly interactions were reduced, and more polymer–solvent interactions were able to occur, resulting in an increase in protein–polymer interactions.

As for the low extension case, PLGA oligomers stayed collapsed across all trials after the restraint was turned off, whereby the frequency of both prot–poly and poly–poly interactions occurring together was much lower when compared to other polymer/ID2S systems. Oligomer aggregation was persistent for some trials, but it did not prevent PLGA/ID2S systems from experiencing irreversible protein–polymer binding (trial 6 at low extension levels), as seen in three out of the seven trials. Moreover, collapsed oligomers did not always aggregate together and stayed in solution. On the contrary, PEG oligomers expanded their structure in the unrestrained phase across simulation trials, regardless of whether they underwent protein contact. Only trials 1 and 3 at low extension levels resulted in irreversible ID2S binding when the restraint was on, likely due to PEG oligomers being free in solution with minimal polymer–polymer interactions occurring. Most protein–polymer binding occurred in the unrestrained phase, since the oligomers exhibit more expanded conformations, as seen with the broader R_g_ probability density distribution. PLGA-PEG/ID2S systems at low extension levels encountered the lowest frequency of protein–polymer interactions across trials, whereby trials 5 and 6 contained oligomers that underwent irreversible binding events. Most oligomers were free in solution and underwent transient prot–poly and poly–poly interactions but, in the unrestrained phase, they did not expand to a great extent and stayed relatively collapsed, similar to PLGA/ID2S systems.

In the medium extension case, oligomers in three PLGA/ID2S trials experienced protein binding but, in other trials, they were mostly dominated by poly–poly interactions or were free in solution. Across all trials, oligomers’ R_g_ decreased quickly to values representative of a collapsed state in the unrestrained phase, even if irreversible binding occurred. In trial 2, however, one oligomer irreversibly bound to ID2S and stayed at the same R_g_ value even after the restraint was turned off, confirming that favorable protein–polymer binding modes are dependent on a polymeric chain’s level of expansion. PEG/ID2S systems at medium extension levels showed oligomers with more frequent prot–poly interactions only in both the restrained and unrestrained phases across trials, in addition to a slight drop in R_g_ and broader R_g_ distribution in the last 100 ns. A low frequency of prot–poly interactions was observed across four PLGA-PEG/ID2S trials, with oligomers being free in solution or undergoing poly–poly interactions. Interestingly, oligomer binding, similar to PLGA/ID2S trial 2, was also observed with PLGA-PEG/ID2S trial 2, whereby an oligomer stayed near the same R_g_ as it was in the restrained phase. Furthermore, some copolymer oligomers in the unrestrained phase did undergo collapse but this was not always the case across trials, unlike the PLGA/ID2S systems; some oligomer R_g_ probability density distributions were similar to those of PEG oligomers at medium extension levels. At the simulated contour lengths used for this study, a medium level of extension, for PEG/ID2S systems, seemed to promote the most frequent prot–poly interactions when the restraint was on; for PLGA/ID2S and PLGA-PEG/ID2S systems, binding events without a drastic collapse in the oligomer structure were observed, indicating that these interactions at a medium level of extension are highly favorable and can overcome dominant LGA self-interactions in the unrestrained phase.

**Figure 5:**
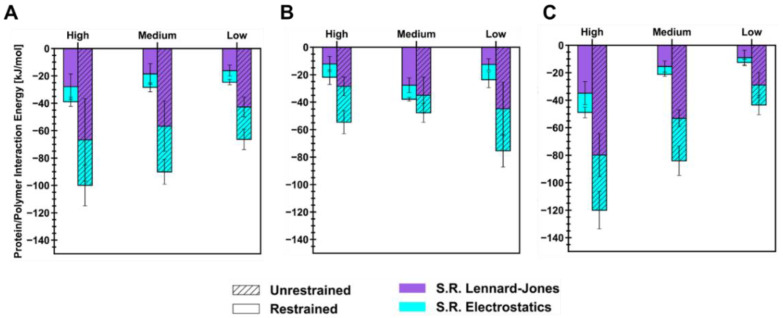
Protein–polymer short-range interaction energies for each level of extension for (**A**) PLGA/ID2S, (**B**) PEG/ID2S, and (**C**) PLGA-PEG/ID2S. For each stacked bar, the sum of Lennard–Jones and electrostatic interaction energies equal the total interaction energy. Mean and its standard error (SE) came from first averaging interaction energies across oligomers (n = 3/trial) then across simulation trials (n = 7).

**Figure A8:**
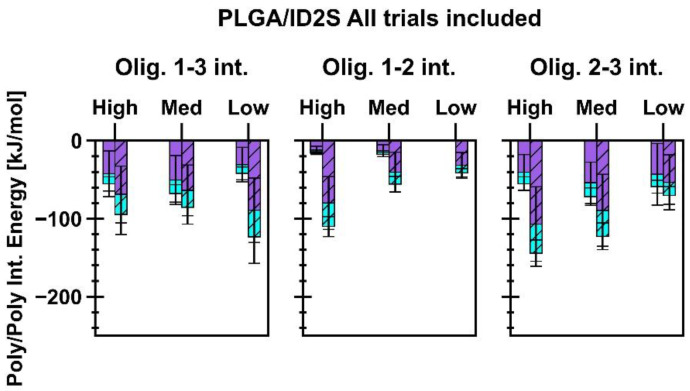

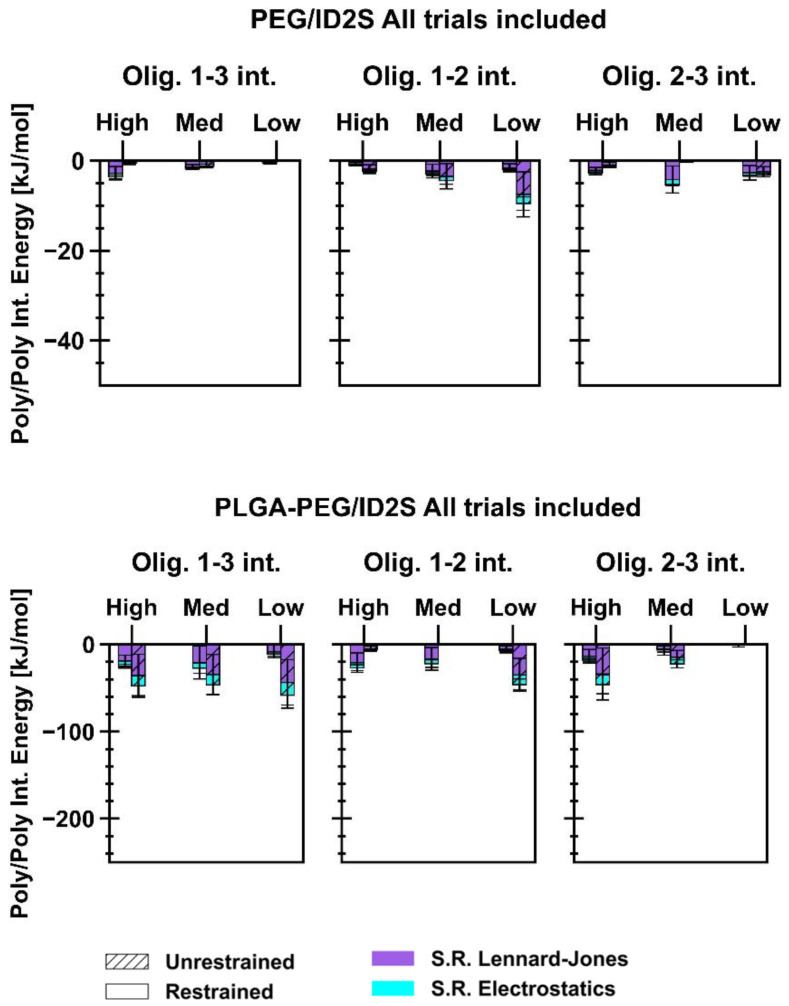
Non-bonded interaction energies for polymer–polymer interactions across different levels of extension for PLGA/ID2S (**top** row), PEG/ID2S (**middle** row), and PLGA-PEG/ID2S (**bottom** row). Mean and SE values were calculated by averaging across trials for each level of extension and restraint phase. Since each simulation contains 3 oligomers, 1–3, 1–2, and 2–3 oligomer interactions were primarily extracted.

Energetics of short-range (SR) non-bonded interactions were extracted from polymer/ID2S simulations to examine the extent of favorable binding between the protein and oligomers, in addition to evaluating oligomer–oligomer behavior. As seen in Figure 5, the total interaction energy (E_int_) across different levels of extension for each polymer type increased after the restraint was turned off, but the magnitude of its increase was seen to be dependent on the extension of the polymer in the restrained phase. This increase can be attributed to the polymer oligomers which interact with more ID2S surface residues, while attempting to reach their equilibrium conformations when dissolved in pure water. For the PLGA/ID2S simulations, a linear decrease in the mean E_int_ was observed as the set oligomer conformation shifted from high to low, with the high extension case showing the most favorable protein–polymer binding. SR Lennard–Jones interaction energies were larger than SR electrostatics across the different levels of extension in both phases, showing that dispersion forces were primarily responsible for PLGA binding. Polymer–polymer interaction energies (Figure A8) were similar in magnitude to protein–polymer interaction energies at high and medium extension levels, showing that expanded structures were important in aiding protein–polymer binding despite the strong attraction between PLGA oligomers. As for the PEG/ID2S systems, a significant increase in E_int_ was seen at high and low extension levels in the unrestrained phase, when compared to the medium level of the extension system with only a slight increase in E_int_. Furthermore, a change in E_int_ in the unrestrained phase was observed to be the largest at the low level of extension, whereas for the high level of extension, E_int_ was similar in magnitude to the medium extension case. This suggests that collapsed PEG oligomers’ return to good solvent conditions, leading to an expansion of chain structure, and resulting in more favorable protein–polymer interactions, when compared to the other level of extension. Polymer–polymer interactions were minimally attractive in nature (Figure A8), further confirming the prevalence of polymer–solvent interactions that drive the increase in R_g_ in the unrestrained phase for PEG oligomers in the low extension case, leading to an increase in more favorable protein–polymer interactions. For highly extended PEG chains, their conformations shifted to lower R_g_ values matching those seen for PEG oligomers in water at the given contour length, allowing them to interact with the ID2S surface at a similar magnitude as the medium level of extension.

The trend in E_int_, observed for the PLGA/ID2S simulations, was the same for the PLGA-PEG/ID2S system. At the highest extension, protein–polymer interactions were most favorable in the restrained and unrestrained phases when compared to medium and low extension levels. The added effect of copolymerization was likely responsible for the observed differences since an expanded PLGA structure in the restrained phase, in addition to PEG domain being in good solvent conditions and PLGA relaxation around the ID2S surface in the unrestrained phase, resulted in overall stronger binding. Moreover, strong polymer–polymer interaction between copolymer oligomers were significantly reduced when compared to PLGA/ID2S systems (Figure A8), further supporting the idea that the PEG domain mediates the extent of LGA interactions and allows for more favorable PLGA–protein binding. Such insights confirm the importance of choosing the right solvent when formulating protein-loaded PLGA-PEG nanoparticles because protein–polymer interactions are likely to be more favorable when PLGA and copolymer domains are highly extended prior to NP formation and growth. Furthermore, neutron scattering measurements of BSA-PEG aqueous mixtures by Abbott et al., showed that PEG had a slightly attractive interaction with BSA, despite having a net repulsive interaction [43]. PEG oligomers have been observed to undergo transient binding with ID2S which is generally less favorable when compared to the other polymer types, corroborating their experimental results.

### 3.2. Characteristics of the Protein–Polymer Interface

**Figure 6:**
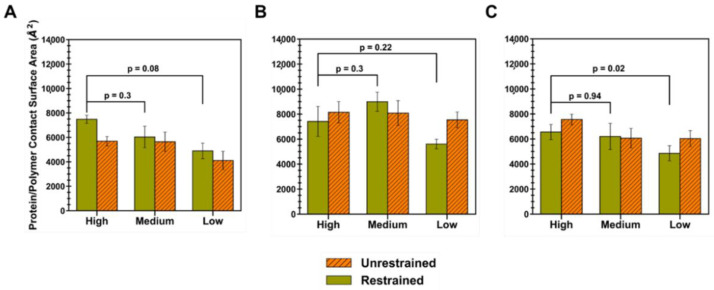
Protein–polymer contact surface area (CSA) at each level of extension for (**A**) PLGA/ID2S, (**B**) PEG/ID2S, and (**C**) PLGA-PEG/ID2S. Mean values and their respective SE came from averaging CSA values of the protein–polymer interface across simulation trials (n = 7). Statistical significance was evaluated using the Wilcoxon signed-rank test, a non-parametric version of the paired T-test that does not assume a normal distribution.

**Figure A9:**
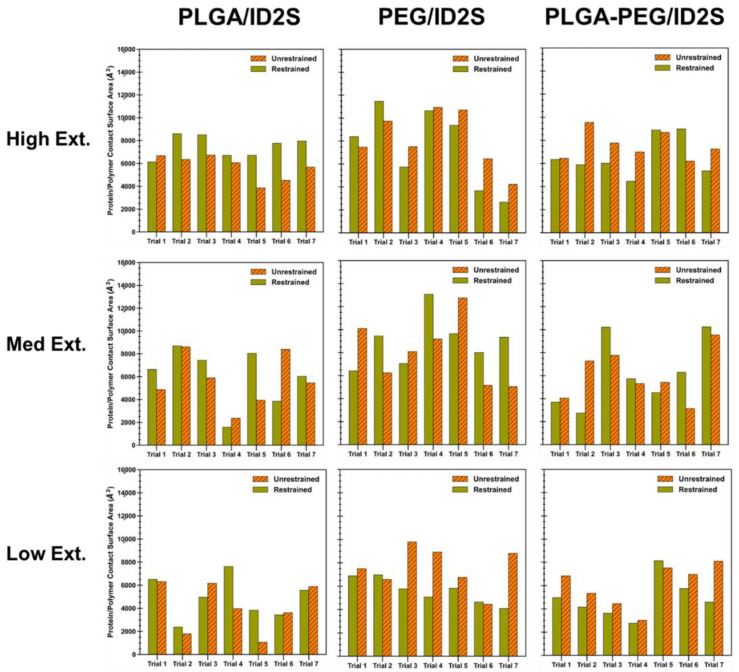
Protein–polymer contact surface area from each simulation trial across the different levels of extension and restraint phases for PLGA/ID2S (**left** column), PEG/ID2S (**middle** column), and PLGA-PEG/ID2S (**right** column). For reference, the ID2S total surface area was 20,438 Å^2^.

In order to understand how the mean interaction surface area varied across polymer types and levels of extension, the protein–polymer CSA was calculated for each simulation trial and averaged, for both the restraint-on and -off phases (Figure 6). Briefly, the CSA is given by Equation (7), where the interface residues are defined by whether a surface residue has a non-zero occupancy value. Out of a total ID2S surface area of 20,438 Å^2^, oligomers at high extension levels, for PLGA/ID2S systems, had the largest average CSA in the restraint-on phase, followed by medium and low extension systems; however, a drop in CSA was observed for the high extension case in the restraint-off phase and across simulation trials, likely due to dominant LGA interactions causing oligomers to exhibit a more semi-collapsed conformation on the ID2S surface. For the restrained phases, the CSA for the high extension case was nearly statistically significant (*p* = 0.08) when compared to the low extension case. A negligible change in CSA was observed for the medium extension case, while a slight drop in CSA was seen for the low extension in the restraint-off phase. The number of trials with CSA values greater than 8000 Å^2^ decreased from three in the medium extension case to zero in the low extension case (Figure A9), showing that expanded PLGA chain conformation results in a larger protein–polymer contact surface area. As for the PEG/ID2S systems, a medium extension of PEG oligomers led to the largest average CSA, when compared to other extension cases. As the restraint is turned off, PEG oligomers contact more of the ID2S surface, as seen with the slight rise in CSA for the high extension case and a greater CSA increase for the low extension case; this was also observed across multiple trials, with the medium extension case in the restraint-on phase resulting in trials with the largest CSA values (>8000 Å^2^) compared to the other polymer types.

For the PLGA-PEG/ID2S systems, high and medium extension cases showed similar CSA values (*p* = 0.94), while the low extension case had a statistically significant lower CSA (*p* = 0.02) when compared to the high extension case in the restrained phase. A majority of trials at high and medium extension levels caused CSA increases in protein–polymer interfaces in the unrestrained phase, but the overall magnitude of the CSA at low extension levels was much lower in both phases when compared to high extension levels. Furthermore, less contact was observed as copolymer oligomers went from medium to low extension levels, similar to the PLGA/ID2S systems. This shows that PLGA-PEG chain expansion in good solvent conditions should allow for greater and more favorable contact with the protein. Moreover, PEG chains moving from a collapsed to semi-extended state resulted in larger protein–polymer contact that was less favorable when compared to PLGA domains. In the context of protein release from a PLGA-PEG nanoparticle in an aqueous environment, PLGA domain collapse in nanoparticle regions where water absorption was significant and LGA interactions were dominant. In addition, PEG–protein interactions were likely not favorable enough to prevent the protein drug from diffusing out of the nanoparticle, reducing the therapeutic efficacy of the delivery vehicle. In addition, protein interactions with collapsed LGA domains would be weak and occur less frequently with a small CSA, causing further drug leakage from the nanocarrier as water diffuses into its matrix. Future drug release studies should consider the relaxation time of the PLGA-PEG polymer at different molecular weights in solvent mediums of interest since a fast transition from a highly extended to collapsed conformation during nanoparticle formation or during drug release could have a critical impact on protein–polymer interactions, which in turn influences protein loading and in vivo release profiles.

**Figure A10:**
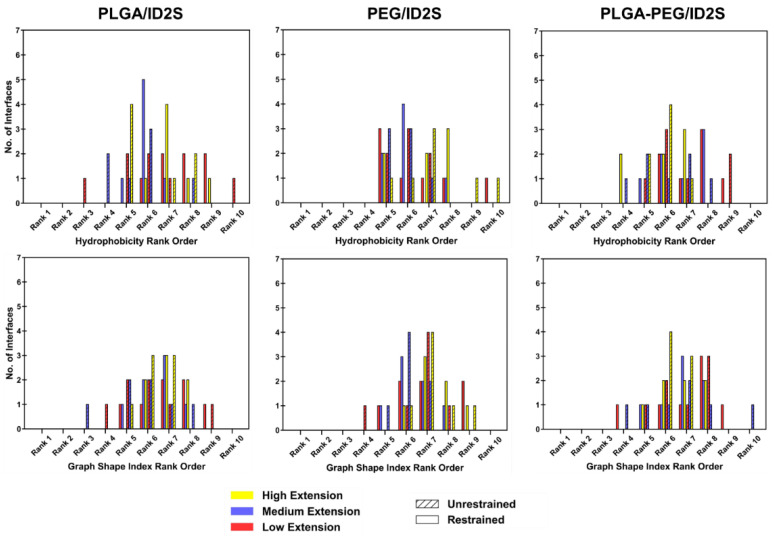
Hydrophobicity and graph shape index rank distributions of the collapsed filtered protein–polymer interface for PLGA/ID2S (**left** column), PEG/ID2S (**middle** column), and PLGA-PEG/ID2S (**right** column) systems.

Physiochemical descriptors of the protein–polymer interfaces, used to calculate CSA for each simulation trial, were ranked relative to those properties for all surface patches to examine how their chemical nature shifts across polymer types. Hydrophobicity, polarizability, normalized van der Waals volume, and the graph shape index of all surface patches were distributed between −0.8–1.4, 0–0.26, 2–5, and 1.4–3.4, respectively, as shown in Figure A5. The graph shape index interface ranks across polymer types and levels of extension were between ranks 4 and 10, with no major discernable trends (Figure A10). As for the hydrophobicity interface rank distribution, most PLGA/ID2S interfaces from the restrained phase at high extension levels had a rank of 7 (hydrophobicity ~0.10), which then shifted to rank 5 (hydrophobicity ~0.16) in the unrestrained phase. This shows that PLGA collapse around the ID2S surface results in the interaction with hydrophobic residues, resulting in a more hydrophobic interface. For the low extension case for both restraint-on and -off phases, a broad interface rank distribution (ranks 3–10) was observed, while a slightly narrower spread (ranks 4–7) was seen for the medium extension case. PEG/ID2S (ranks 5–8) and PLGA-PEG/ID2S (ranks 4–9) interfaces also showed the same widespread expression and did not show any major differences across the levels of extension.

**Figure A11:**
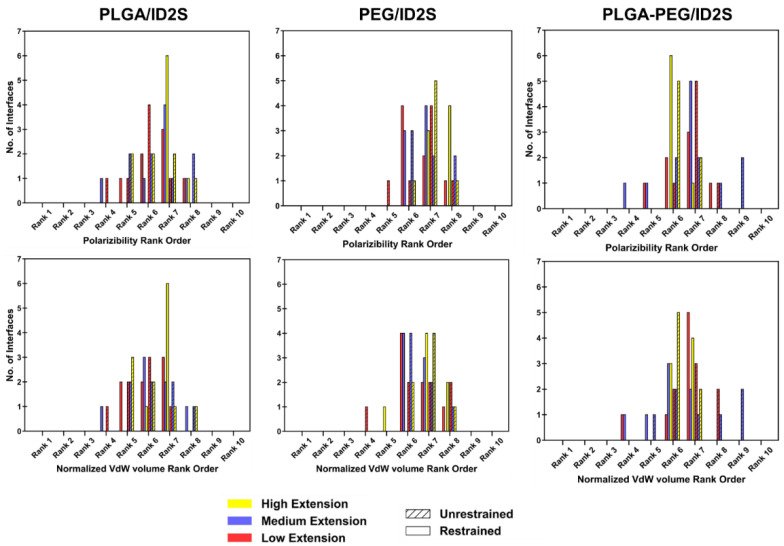
Polarizability and normalized van der Waals volume rank distributions of the collapsed filtered protein–polymer interface for PLGA/ID2S (**left** column), PEG/ID2S (**middle** column), and PLGA-PEG/ID2S (**right** column) systems.

Polarizability and normalized van der Waals volume (Figure A11), on the other hand, were more valuable in understanding variations in the physiochemical properties of the protein–polymer interface. At high extension levels, the PLGA/ID2S interface ranks for polarizability were centered at ranks 7–8 (polarizability ~0.14) in the restrained phase but shifted to a broader rank spread (ranks 4–8) in the unrestrained phase, indicating that oligomers also interacted with more polarizable residues during their contraction around ID2S. At low and medium levels of extension, polarizability ranks were between 4 and 8, with a peak at ranks 6–7. For PEG/ID2S interfaces, polarizability ranks were centered between ranks 6 and 8 across different extension levels, whereas for the PLGA-PEG/ID2S interfaces, polarizability ranks at high extension fell mostly on rank 6 in the restrained phase but shifted to ranks 3–10 in the unrestrained phase, similar in trend to PLGA/ID2S interface ranks; this could be due to PEG domains interacting with more ID2S surface residues in some simulation trials after the restraint is off. Lastly, normalized van der Waals volume parameter ranking for PLGA/ID2S at high extension levels showed six out of seven interfaces, from the restrained phase, which had a rank of 7 (a normalized van der Waals volume of ~3.3), whereas some interfaces from the unrestrained phases changed to ranks 5–6; such differences indicate that the PLGA oligomers contact more residues with a slightly larger normalized van der Waals volume when released from the restraint. At low and medium extension levels, normalized van der Waals volume ranks were distributed between 4 and 8, regardless of restraint-on or -off phases. PEG/ID2S (ranks 4–8) and PLGA-PEG (ranks 4–9) interfaces shows a similar spread in the normalized van der Waals volume ranking across levels of extension and in both phases, with peaks observed between ranks 6 and 7. Overall, shifts in parameter ranking distribution for the protein–polymer interfaces were useful in the identification of unique interface characteristics, in addition to understanding how the unrestrained phase results in PLGA/ID2S interfaces are more polarizable, larger in their van der Waals volume, and slightly more hydrophobic.

**Figure 7:**
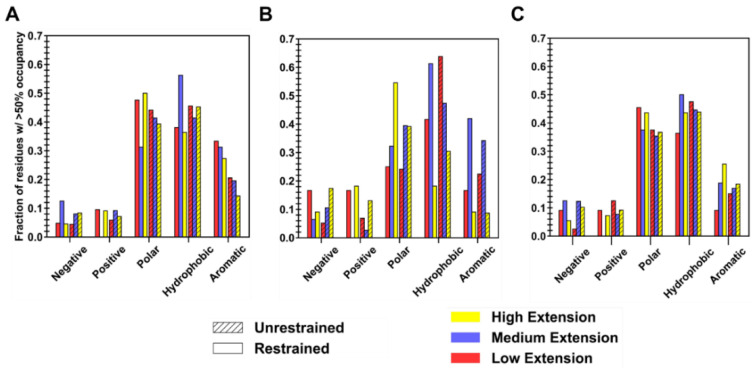
Fraction of residues, constituting the averaged collapsed interface (n = 7) from both the restraint-ON and -OFF phase, with occupancy values greater than 50% at different levels of oligomer extension for (**A**) PLGA/ID2S (**B**) PEG/ID2S, and (**C**) PLGA-PEG/ID2S systems. Amino acids were grouped into 5 respective categories: negative, positive, polar, hydrophobic, and aromatic, where aromatic residues were also counted in the hydrophobic group.

**Figure A12:**
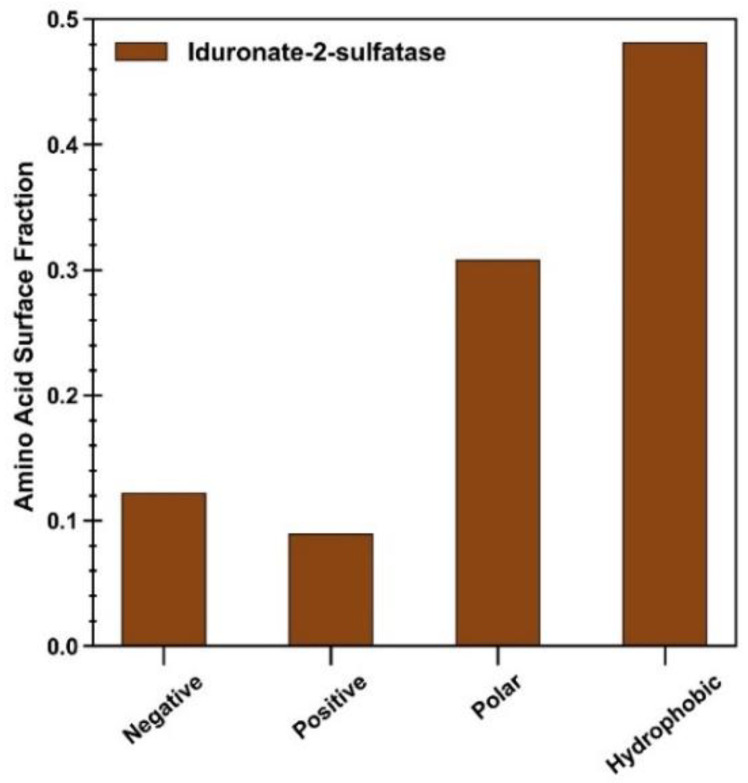
Surface amino acid composition of iduronate-2-sulfatase at pH 7 in its native state. Surface residues consisted of 393 out of the 516 total residues.

Surface residues that are responsible for favorable binding for each polymer type were extracted by first generating a collapsed protein–polymer interaction interface from all simulation trials in both restraint-on and -off phases (two averaged interfaces per level of extension), containing amino acids with a non-zero percent occupancy. A cutoff of >50% was then applied to each collapsed interface to sub-select high-residency residues, whose amino acid composition can be seen in Figure 7. For clarity, high-residency residues are amino acids with >90% occupancy, while residues with occupancies between 50 and 90% are considered to be important in constructing the protein–polymer interface. Given that ID2S surface residues are composed of ~30% polar, ~48% hydrophobic, ~12% negatively, and ~9% positively charged residues at neutral pH in its native state (Figure A12), it is not surprising that a large fraction of residues are primarily polar and hydrophobic, with fractional values being less than 0.15 for charged surface residues across polymer types. The ID2S-PLGA filtered interface, in the restrained phase at high and low extension levels, was primarily composed of mostly polar and hydrophobic residues, where aromatic groups contribute a significant proportion to the fraction of nonpolar residues that drive binding. In the unrestrained phase, the fraction of hydrophobic residues increases while that of polar and aromatic residues decreases, suggesting that PLGA oligomers increased their interactions with non-aromatic hydrophobic residues as they collapsed around the ID2S surface. As for the medium extension case when the restraint was on, the hydrophobic fraction of residues was the largest (~0.6), while polar and aromatic fractions were similar in value, indicating that this intermediate R_g_ value was highly favorable for hydrophobic interactions to occur between PLGA and ID2S. In the restraint-off phase, an increase in the polar fraction was observed along with a decrease in hydrophobic and aromatic fraction, showing that both polar and hydrophobic residues drive PLGA oligomer stabilization on the ID2S surface; the presence of methyl and carboxyl groups within polymer chain likely mediate favorable contact with ID2S, resulting in large occupancy values and favorable polymer binding.

As for PEG/ID2S collapsed interface in the restrained phase at high extension levels, polar residues made up a large portion, followed by positively charged and hydrophobic residues; in the unrestrained phase, the fraction of polar and positively charged residues dropped while an increase in the fraction of negatively charged and hydrophobic residues was observed. This could be due to the shift in PEG oligomers toward ideal-chain conformations, resulting in a greater contact with non-aromatic hydrophobic residues. At low extension cases in the restrained phase, charged and aromatic residues made up a similar fraction (~0.15), with the rest of the interface being composed of hydrophobic residues. The polar fraction of residues remained constant in the unrestrained phase, while a drastic increase in the hydrophobic residues fraction and decrease in the charged residue fraction were observed. Such trends indicate an increase in ID2S/PEG CSA in the unrestrained phase is due to expanded PEG oligomers contacting a greater number of hydrophobic residues. At medium extension levels, polar and hydrophobic residues composed a large fraction of the collapsed interface in both restraint-on and -off phases, with a slight decrease and increase in the polar fraction being seen in the unrestrained phase; this can be explained by the strength of polymer–solvent interactions that pulls PEG oligomers away from the ID2S surface and allows them to explore other polar residues.

At high extension levels for the PLGA-PEG/ID2S interfaces, polar and hydrophobic residue fractions combined to be ~80%, for the restrained phase, but polar and aromatic residue fraction decreased, while the hydrophobic fraction stayed constant for the unrestrained phase. This could be due to persistent PLGA-ID2S interactions and PEG’s slight attraction to ID2S, across the various simulation trials. Polar and negatively charged fraction of residues decreased when compared to interfaces from both phases in the low extension case, while polar and hydrophobic fraction increased. For the medium extension case, a slight decrease for the hydrophobic and polar categories was observed, as well as an increase in positively charged fraction, indicating that irreversible copolymer-ID2S binding could be attributed to polar residues, in addition to a mixture of aromatic and non-aromatic hydrophobic AAs. Overall, the interplay of chemical moieties on a polymer chain and its time-averaged conformation in solution have a critical impact on whether strong protein–polymer binding events occur during nanoparticle formulation or degradation.

### 3.3. Surface Patch Characteristics at High and Low Extension Levels

**Figure 8:**
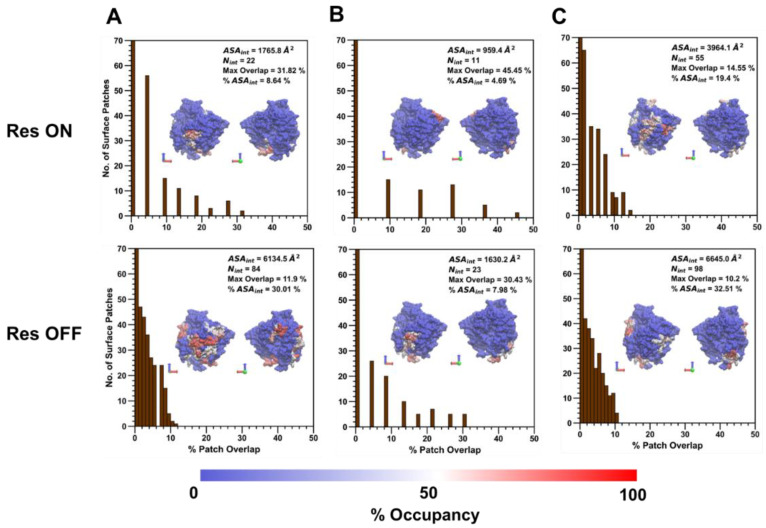
At high extension levels, the distribution of the number of contiguous surface patches that overlap with the collapsed averaged protein–polymer interface containing residues with >50% occupancy, at various overlap percentages, is shown for (**A**) PLGA/ID2S, (**B**) PEG/ID2S, and (**C**) PLGA-PEG/ID2S. Within each plot is the visualization of the collapsed interface, whereby red indicates max occupancy and blue indicates no occupancy. ASA_int_ is the surface area of the collapsed prot–poly interface, N_int_ is the total number of residues in the collapsed interface, and % ASA_int_ is the percentage of ID2S surface area that is composed of the prot–poly collapsed interface. Max overlap value for each polymer/ID2S interface is also shown in each plot above.

Following the analysis of the amino acid composition of the collapsed protein–polymer interface containing residues with >50% occupancy across polymer types, the percent patch overlap of generated surface patches with the polymer/ID2S interface was calculated for the high (Figure 8) and low (Figure 10) extension cases to show how residue clusters containing high-residency surface AAs can be identified using this patch analysis method proposed by Jones et al. [39,44]. Across all polymer/ID2S systems, most surface patches had an overlap with the collapsed interface between 0 and 1%, with ~8–15% of patches having an overlap greater than 5%; moreover, N_int_ was smaller in the restraint-on phase, when compared to the restraint-off phase, across all polymer types. This is likely due to the presence of the restraint ensuring that the polymer oligomers maintain the set R_g_; thus, monomers that are weakly bound to the ID2S surface will be pulled off, resulting in a large majority of interacting surface residues with low occupancy values. This idea is also supported by an analysis of non-bonded interaction energetics and the smaller ASA_int_ of the filtered interface, when compared to the average CSA for polymer/ID2S systems at high extension levels for the restrained phase. As N_int_ increases for the unrestrained phases, a general drop in the percent max overlap for certain surface patches was observed. For the PLGA/ID2S filtered interface, the decrease in the percent max overlap from 31.8% to 11.9% signifies that the increase in the interface area results in a fewer amount of contiguous surface patches that overlap with a high percentage of the protein–polymer interface. Regardless, most surface patches with the largest percent overlap typically contained high-residency residues. As for the PEG/ID2S interfaces, ASA_int_ and N_int_ were smaller in magnitude than the other polymer/ID2S interfaces, providing more evidence that PEG–water interactions were dominant and PEG oligomers preferred to be in solution; furthermore, % max overlap only dropped to 30.4%. The effect of high extension and copolymerization can also be seen in the PLGA-PEG/ID2S interface, whereby ASA_int_ and N_int_ were largest and % max overlap values were the smallest in both restraint-on and -off phases when comparing to the homopolymer/ID2S systems.

**Figure 9:**
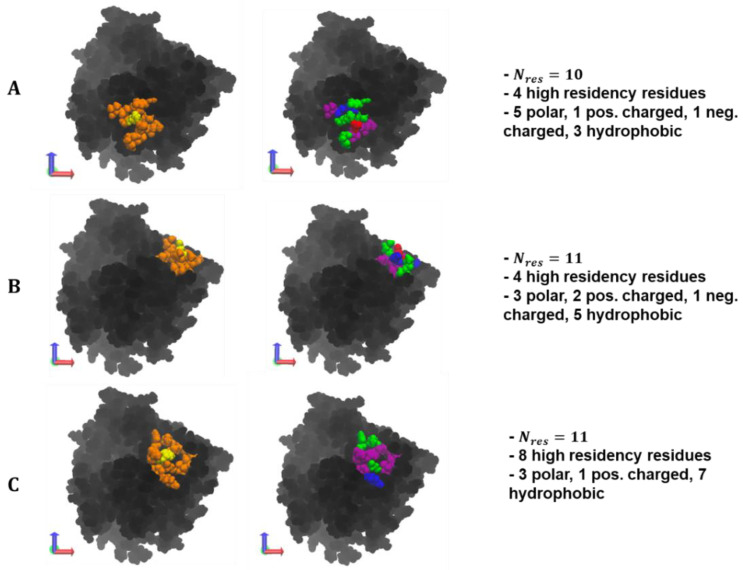
At high extension levels, an individual surface patch with the highest patch overlap percentage with the filtered interface is shown from (**A**) PLGA/ID2S Res OFF phase, (**B**) PEG/ID2S Res ON phase, and (**C**) PLGA-PEG/ID2S Res ON phase. In the first column of images, yellow signifies the central residue from which the patch was generated, and orange indicates residues that were included in the given patch by satisfying the θ_cut_ cutoff requirement of 125°. Amino acid composition of each patch can be seen in the second column of images, where blue residues are positively charged, red residues are negatively charged, green residues are polar, and purple residues are hydrophobic. N_res_ is the number of residues in the selected patch.

A visualization of the max overlap patches and the amino acid composition can be found in Figure 9. Across the polymer types, selected residue clusters consisted of 10–11 residues, with a majority of them being polar and hydrophobic and varying in shape and morphology. The selected surface patches, across the polymer/ID2S systems, contained four and eight high-residency residues, showing that this method is capable of identifying residue clusters containing polymer binding hotspots. For the PLGA/ID2S patch, the distance between the three hydrophobic residues, filled with the five polar residues, could result in a binding mode where carboxyl groups on the oligomer chain first undergo dipole–dipole interactions then as the oligomer settles on the ID2S surface, methyl groups on either side of the chain experience hydrophobic interactions, leading to irreversible binding. The PEG patch had three charged and three polar residues, suggesting that this residue cluster would be relatively hydrophilic and would undergo hydrogen bonding or dipole–dipole interactions with PEG oligomers; hydrophobic residues were interspersed within the patch and provided some hydrophobic character. Seven hydrophobic residues covered most of the PLGA-PEG patch, along with the three polar residues and one positively charged residue. The morphology of this residue cluster is likely to promote binding of the PLGA domain and allow for oligomer collapse, since multiple methyl groups can interact with the hydrophobic strip as the chain changes conformation. Overall, selected patches at the highest percent overlap with the prot–poly interaction interface allows for the systematic identification of binding zones that can be evaluated for their uniqueness and be engineered to increase nanoparticle drug loading or tune drug release profiles.

**Figure 10:**
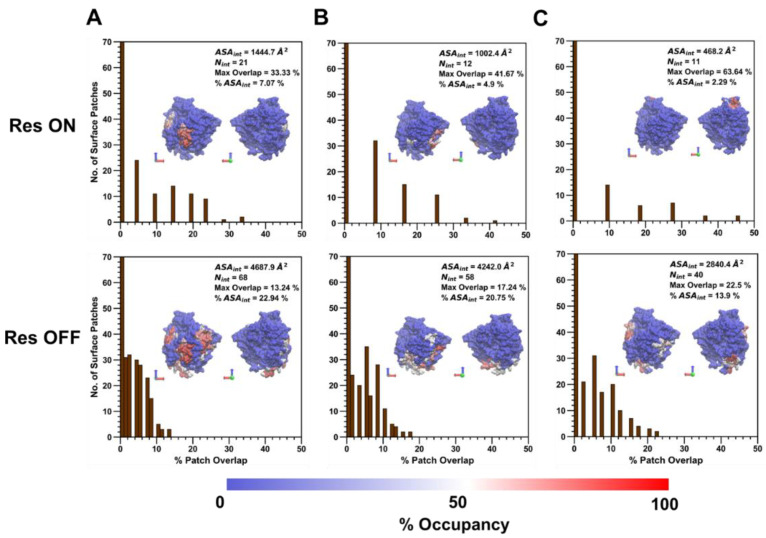
At low extension levels, the distribution of the number of contiguous surface patches that overlap with the collapsed averaged protein–polymer interface containing residues with >50% occupancy, at various overlap percentages, is shown for (**A**) PLGA/ID2S, (**B**) PEG/ID2S, and (**C**) PLGA-PEG/ID2S. Within each plot is the visualization of the collapsed interface, whereby red indicates the max occupancy and blue indicates no occupancy. ASA_int_ is the surface area of the collapsed prot–poly interface, N_int_ is the total number of residues in the collapsed interface, and % ASA_int_ is the percentage of ID2S surface area that is composed of prot–poly collapsed interface. Max overlap value for each polymer/ID2S interface is also shown in each plot above.

PEG/ID2S and PLGA-PEG/ID2S filtered interfaces in the restrained phase had smaller ASA_int_ and N_int_ values when compared to the PLGA interface (Figure 10). Moreover, the percent max overlap of certain surface patches increased as the interface became smaller, when going from PLGA, PEG, and then the copolymer. This shows that the PEG and copolymer oligomers at low extension levels undergo mostly reversible binding with ID2S and tend to be free in solution or interact with another oligomer in solution. PLGA oligomers in both the restrained and unrestrained phase, however, were still able to undergo favorable and irreversible binding with 7% and 22.9% of the protein surface area, even in the presence of strong polymer–polymer interactions. Comparing to the PLGA interface surface area at high extension levels from the restraint-off phase, ASA_int_ and N_int_ were smaller, showing that expanded PLGA oligomers lead to interactions with more surface residues. The PLGA/ID2S interface percent max overlap was the lowest among the polymer types for interfaces from the restraint-off phase. When the restraint was turned off, the large increase observed in ASA_int_ and N_int_, in addition to the drop in percent max overlap for the PEG/ID2S interface, further confirms the oligomers’ return to good solvent conditions from a collapsed conformation results in an increased interaction with ID2S surface residues. A similar trend was also seen for PLGA and PLGA-PEG interfaces. In this case, the collapsed PLGA domain mostly interacted with hydrophobic and polar residues, as does the PEG domain in the scenario of ideal-chain conditions. These observations help explain why there was an increase in CSA from the unrestrained phase.

**Figure 11:**
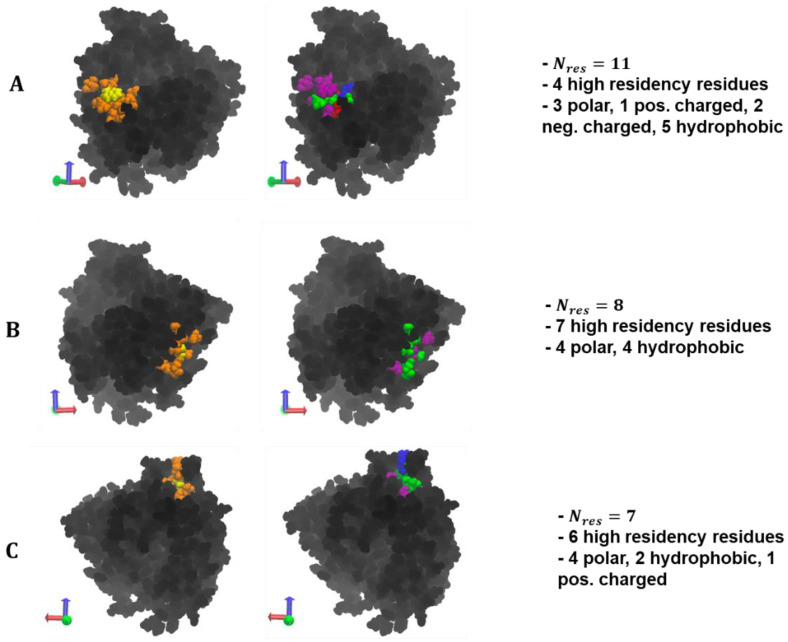
At low extension levels, an individual surface patch with the highest patch overlap percentage with the filtered interface is shown from (**A**) PLGA/ID2S Res OFF phase, (**B**) PEG/ID2S Res ON phase, and (**C**) PLGA-PEG/ID2S Res ON phase. In the first column of images, yellow signifies the central residue from which the patch was generated, and orange are residues that were included in the given patch by satisfying the θ_cut_ cutoff requirement of 125°. Amino acid composition of each patch can be seen in the second column of images, where blue residues are positively charged, red residues are negatively charged, green residues are polar, and purple residues are hydrophobic. N_res_ is the number of residues in the selected patch.

For the PLGA 11-residue patch at low extension levels (Figure 11), hydrophobic residues surround polar residues and charged residues seemed to be slightly less exposed to the surface. Collapsed oligomers with methyl groups on their surface were likely able to favorably contact the front-facing hydrophobic residues, leading to irreversible binding. As for the PEG patch, four polar and four hydrophobic residues primarily make up this eight-residue cluster, whereby seven surface residues had high occupancy values. This may be due to the spread of polar residues that allow for multiple opportunities for hydrogen bonding, while hydrophobic residues interact with methylene groups on the PEG chain. The seven-residue patch for the copolymer interface was made up of four polar and two hydrophobic AAs, with one positively charged residue. Comparing to the location of high-residency residues for PLGA-PEG/ID2S interface in the restraint-on phase, it is reasonable to conclude that this patch and its arrangement of polar and hydrophobic residues resulted in favorable copolymer binding, driven by the presence of methyl, carboxyl, and/or ester groups. It should be noted that these selected surface patches also promoted strong oligomer binding across the polymer types at both levels of extension. A stricter occupancy cutoff can be employed to generate a smaller protein–polymer interface, leading to larger % max overlap values for certain surface patches. Ultimately, an examination of the local chemical environment of the protein surface with strong protein–polymer binding is a nontrivial task, and this patch analysis provides a way to gain such information.

## 4. Conclusions

In this work, PLGA, PEG, and PLGA-PEG oligomers were simulated in the presence of ID2S at various level of extension to examine the role of polymer conformation on protein–polymer interactions. This enables greater mechanistic understanding into the molecular-level driving forces that are present during protein loading within PLGA-PEG nanoparticles and during nanoparticle dissolution, leading to protein release. Highly expanded PLGA-PEG conformations were shown to lead to greater ID2S contact and subsequent movement to poor solvent conditions, by turning off the restraint, resulted in persistent binding, even in the presence of strong LGA-LGA interactions. Moreover, collapsed LGA domains can still favorably and irreversibly interact with ID2S. This suggests that relaxation times of the PLGA homopolymer and PLGA-PEG copolymer at different molecular weights in solvent mediums relevant to drug release studies should be considered. PEG oligomers’ weak attraction to proteins, in addition to dominant LGA interactions, could explain why a burst release profile may be observed for some protein-loaded PLGA-PEG nanoparticles. As water and ions infiltrate the nanoparticle matrix, PEG domains begin to transition to ideal-chain conformations. In addition, the degradation of large PLGA domains into smaller oligomers results in their collapse into LGA-rich parts of the nanoparticle matrix, leading to a decrease in protein–polymer interactions. Since the PEG shell prefers to be in the aqueous solvent and as ions promote the further hydration of proteins, released proteins will be able to diffuse into the surrounding solvent medium and be detected. Overall, the successful encapsulation of protein drugs within PLGA-PEG nanoparticles and their administration in vivo will require protein–polymer interactions that can match or outcompete the strength of LGA self-interactions, in order to ensure the proper shielding of therapeutic proteins from the surrounding environment.

## Figures and Tables

**Table 1:** The monomer length (N_mon_) and contour length (L_c_) of the 3 oligomers, for each polymer type, simulated in this study. Each PLGA-PEG oligomer consisted of N_LGA_ = 12 and N_EG_ = 13. This was performed to ensure that the simulated oligomers were still diblock copolymers with a nearly 1:1 LGA:EG ratio at the given contour length.

Polymer	N_mon_	L_c_ [nm]
PLGA	20	10.5
PLGA-PEG	25	10.4
PEG	33	10.4

## Data Availability

Please email Chris W. Nyambura at cnyambr95@gmail.com or Jim Pfaendtner at jpfaendt@uw.edu to gain access to the generated data used in this study.
